# Supplementary material for: Developmental prosopagnosics have normal spatial integration in posterior ventral face-selective regions
Source: bioRxiv. 2025 Jul 26:2025.07.25.666588. Preprint. [Version 1] doi: 10.1101/2025.07.25.666588 (PMC12330551; doi:10.1101/2025.07.25.666588)
Supplement: 1 [file NIHPP2025.07.25.666588V1-supplement-1.pdf]

bioRxiv preprint doi: https://doi.org/10.1101/2025.07.25.666588; this version posted July 26, 2025. The copyright holder for this preprint (which was not certified by peer review) is the author/funder, who has granted bioRxiv a license to display the preprint in perpetuity. It is made available under aCC-BY 4.0 International license.

Supplementary Figures and Tables

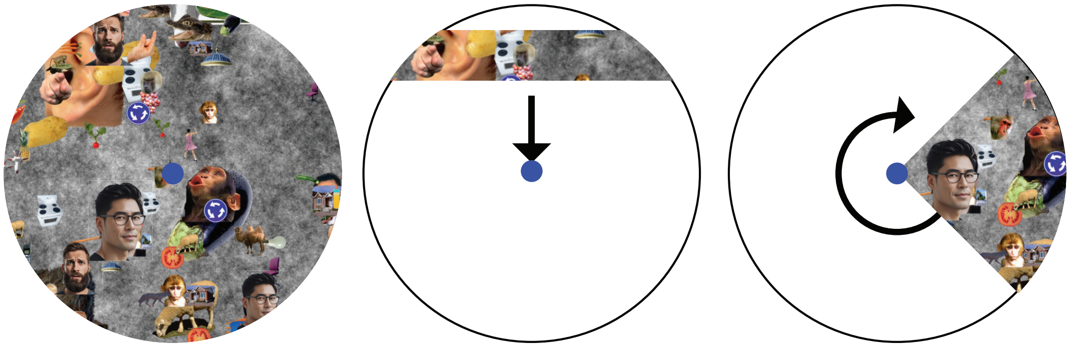

Appendix 0—figure 5. Schematic diagram of retinotopic mapping runs. Diagram is for illustration purposes only and not drawn to scale. All original faces have been replaced with AI-generated faces to preserve identifying information of original people. Runs began with a 4 second full-field exposure followed by eight sweeps of bar stimuli in eight different directions and ending with a single clockwise rotation of a wedge. Participants fixated a central dot throughout each run and indicated via button press whenever the dot changed color to red. The carrier images shown inside each aperture changed every 250 msecs (5 Hz), alternating between natural outdoor scenes and collages of various visual objects on backgrounds of pink noise (shown). See Methods for more details.

bioRxiv preprint doi: https://doi.org/10.1101/2025.07.25.666588; this version posted July 26, 2025. The copyright holder for this preprint (which was not certified by peer review) is the author/funder, who has granted bioRxiv a license to display the preprint in perpetuity. It is made available under aCC-BY 4.0 International license.

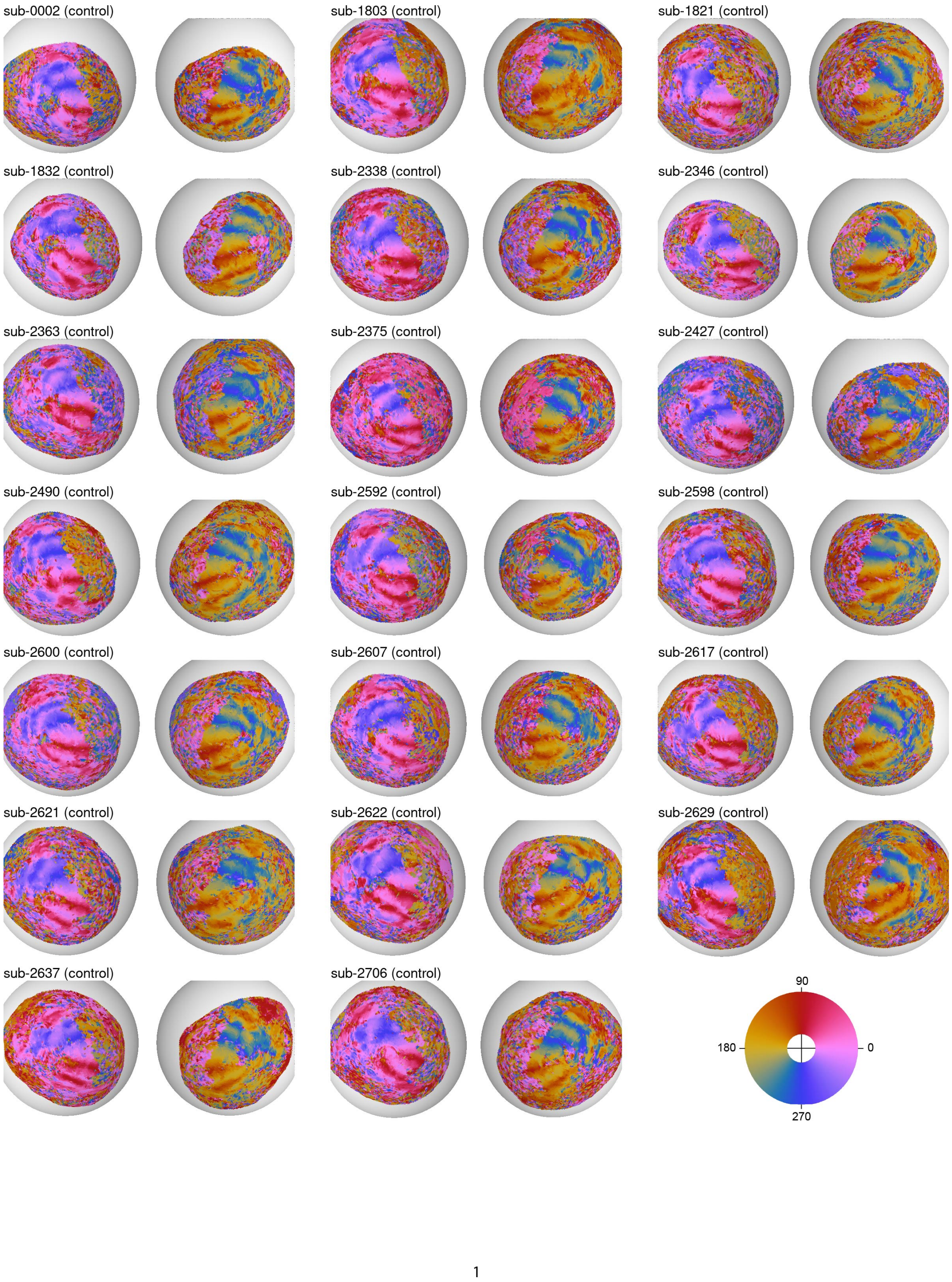

Appendix 0—figure 6. Polar angle plots displayed on inflated cortical surfaces for all control participants.

bioRxiv preprint doi: https://doi.org/10.1101/2025.07.25.666588; this version posted July 26, 2025. The copyright holder for this preprint (which was not certified by peer review) is the author/funder, who has granted bioRxiv a license to display the preprint in perpetuity. It is made available under aCC-BY 4.0 International license.

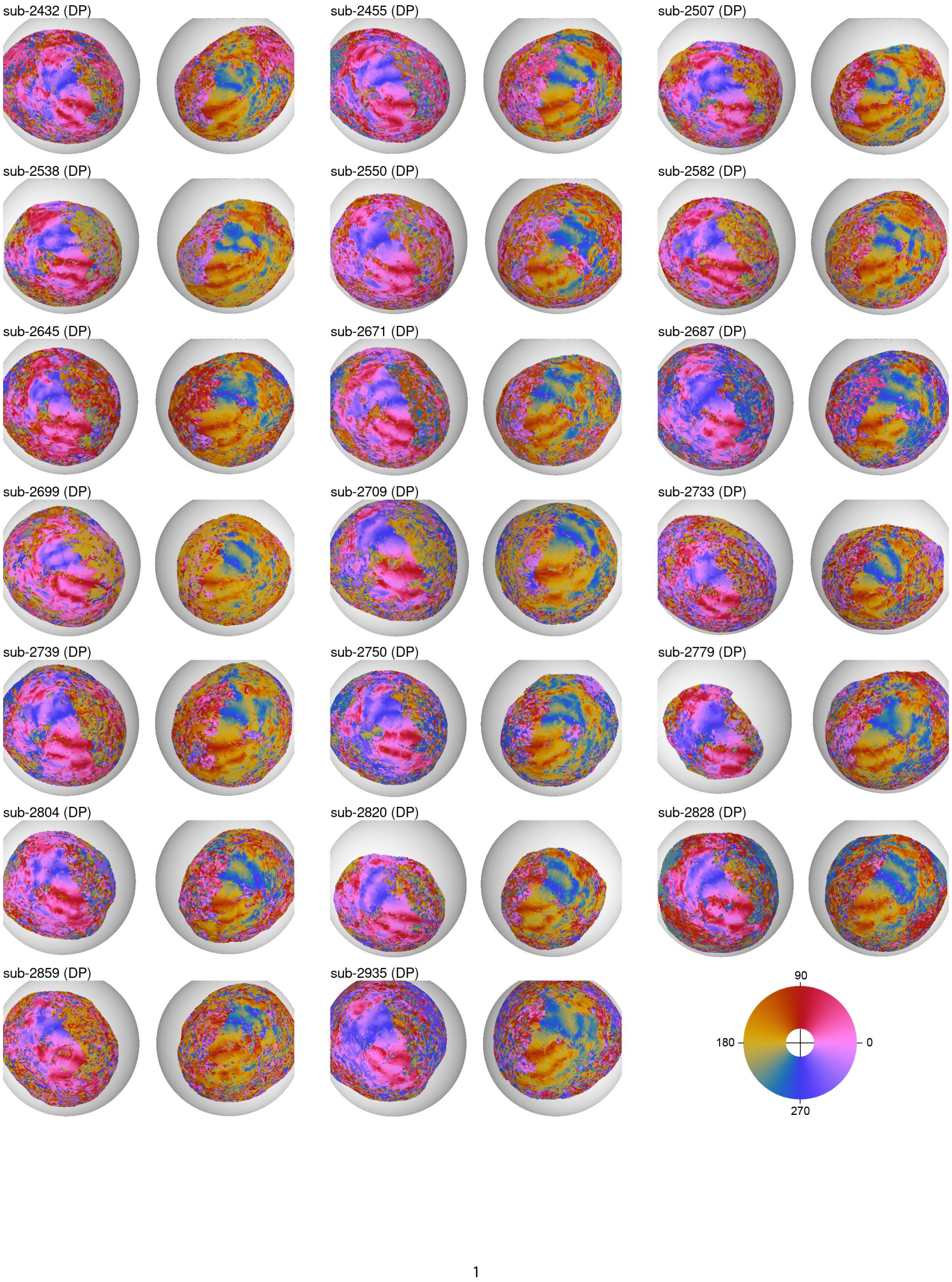

Appendix 0—figure 7. Polar angle plots displayed on inflated cortical surfaces for all DP participants.

bioRxiv preprint doi: https://doi.org/10.1101/2025.07.25.666588; this version posted July 26, 2025. The copyright holder for this preprint (which was not certified by peer review) is the author/funder, who has granted bioRxiv a license to display the preprint in perpetuity. It is made available under aCC-BY 4.0 International license.

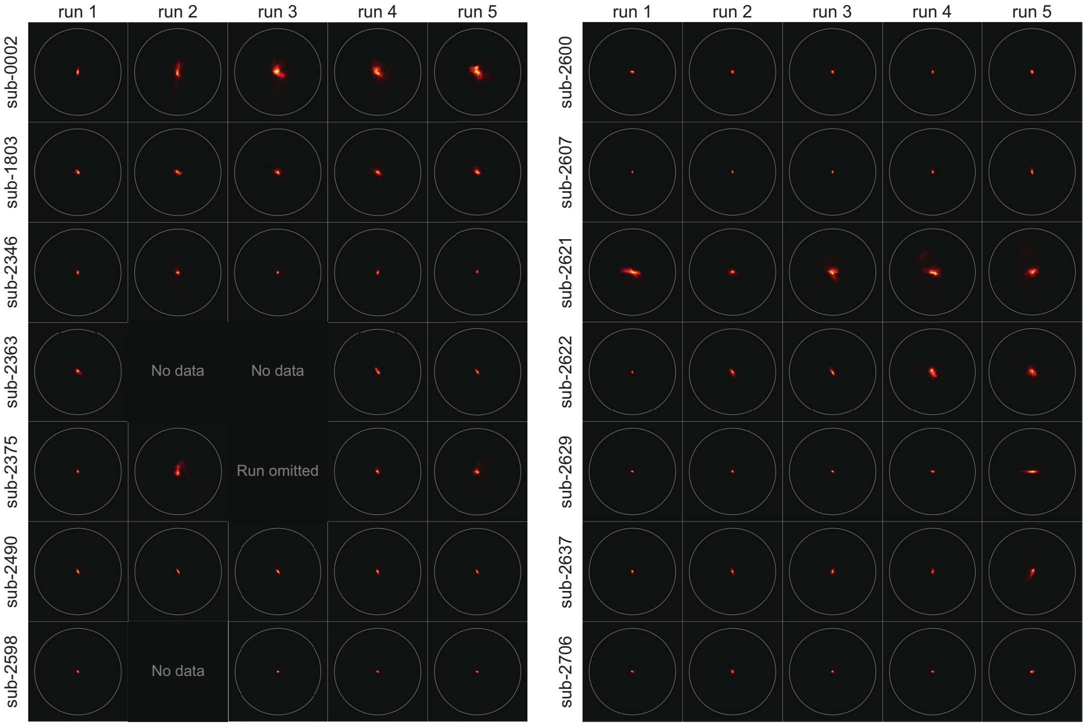

Appendix 0—figure 8. Two dimensional density plots (heatmaps) of eye position recordings by subject (controls) and run.

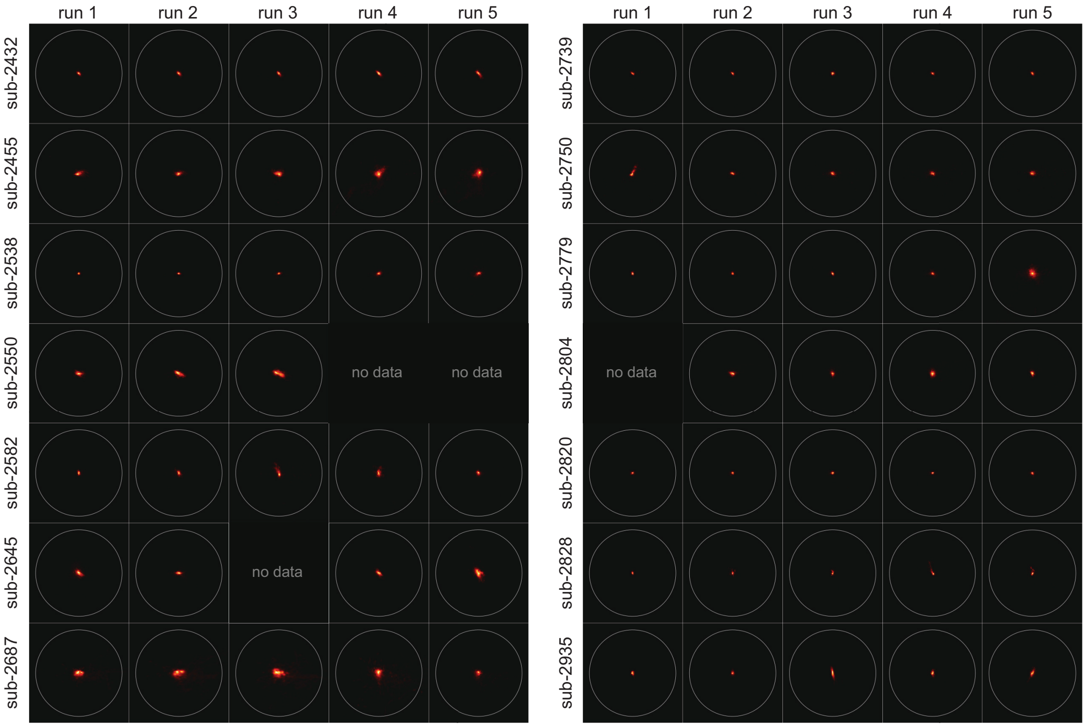

Appendix 0—figure 9. Two dimensional density plots (heatmaps) of eye position recordings by subject (DPs) and run.

bioRxiv preprint doi: https://doi.org/10.1101/2025.07.25.666588; this version posted July 26, 2025. The copyright holder for this preprint (which was not certified by peer review) is the author/funder, who has granted bioRxiv a license to display the preprint in perpetuity. It is made available under aCC-BY 4.0 International license.

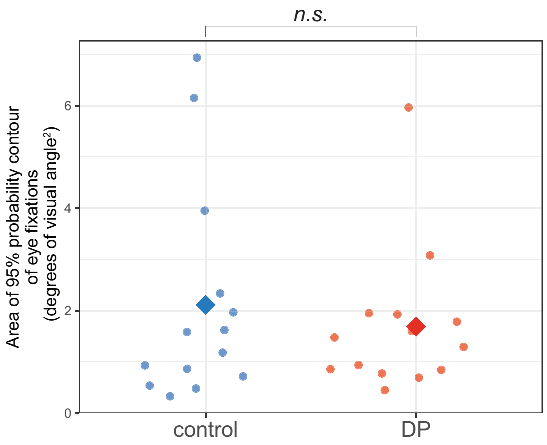

Appendix 0—figure 10. Eye fixation performance during the retinotopic mapping experiment for controls and DPs. Eye fixation performance was summarized by computing the area of the 95% probability contour of eye position samples. Points are averages across all runs obtained. Diamonds are averages for each group (Controls, DPs).

bioRxiv preprint doi: https://doi.org/10.1101/2025.07.25.666588; this version posted July 26, 2025. The copyright holder for this preprint (which was not certified by peer review) is the author/funder, who has granted bioRxiv a license to display the preprint in perpetuity. It is made available under aCC-BY 4.0 International license.

		Faces (PSC)		Objects (PSC)					Cohen's *d*		
ROI	hemisphere	Control	DP	Control	DP	*t*	df	*p*	lower	effect	upper
OFA	right	2.33±0.08	2.09±0.07	1.08±0.06	1.27±0.06	3.28	34.75	0.002*	-1.72	-1.06	-0.39
OFA	left	1.72±0.09	1.51±0.06	0.89±0.06	1.06±0.06	2.87	32.36	0.007*	-1.58	-0.93	-0.27
pFUS	right	2.65±0.08	2.42±0.07	1.24±0.08	1.25±0.05	1.56	35.32	0.1280	-1.13	-0.51	0.13
pFUS	left	2.13±0.06	1.81±0.07	1.09±0.06	1.19±0.05	3.06	36.09	0.004*	-1.64	-0.99	-0.33
mFUS	right	2.06±0.07	1.62±0.08	0.64±0.05	0.63±0.04	2.36	37.84	0.0240	-1.40	-0.77	-0.12
mFUS	left	1.62±0.08	1.31±0.05	0.56±0.04	0.5±0.03	1.70	28.41	0.1000	-1.18	-0.55	0.08

Appendix 0—table 3. Face-selectivity was defined as the percent signal change to blocks of face stimuli minus the percent signal change to blocks of object stimuli. To quantitatively compare face-selectivity in DPs and controls, we used the variable window method (Norman-Haignere et al., 2013) whereby voxels were selected for the analysis by applying group-defined region of interest (ROI) masks and choosing the top 20% of the voxels with the highest t value for the faces-minus-objects contrast. Voxel selection was fully cross-validated in a leave-one-run-out fashion – voxels were selected based on three out of four runs and face selectivity was measured from the left out run. For each participant, the final measure of face-selectivity was the average across four cross-validated folds. For each ROI and hemisphere, separate Welche two sample t-tests were conducted comparing data from DPs to controls. Due to the number of tests conducted, a more conservative alpha threshold of $p < 0.01$ was used to establish statistical significance.
$* \ p < 0.01$; $** \ p < 0.001$

bioRxiv preprint doi: https://doi.org/10.1101/2025.07.25.666588; this version posted July 26, 2025. The copyright holder for this preprint (which was not certified by peer review) is the author/funder, who has granted bioRxiv a license to display the preprint in perpetuity. It is made available under aCC-BY 4.0 International license.

	Estimate	*SE*	df	*t*	*p*
**V1**					
(Intercept)	45.50	1.43	49.27	31.91	0.000
group (DP)	-2.09	2.02	49.28	-1.04	0.305
hemisphere (right)	-0.85	0.93	40.02	-0.92	0.362
group:hemisphere	0.82	1.31	40.03	0.63	0.535
**V2**					
(Intercept)	46.85	1.43	49.48	32.77	0.000
group (DP)	-0.04	2.02	49.49	-0.02	0.984
hemisphere (right)	1.70	0.94	39.86	1.81	0.078
group:hemisphere	-0.42	1.33	39.89	-0.32	0.754
**V3**					
(Intercept)	46.85	1.43	49.48	32.77	0.000
group (DP)	-0.04	2.02	49.49	-0.02	0.984
hemisphere (right)	1.70	0.94	39.86	1.81	0.078
group:hemisphere	-0.42	1.33	39.89	-0.32	0.754
**hV4**					
(Intercept)	50.66	2.19	53.36	23.13	0.000
group (DP)	-0.02	3.10	53.36	-0.01	0.995
hemisphere (right)	2.54	1.69	39.85	1.51	0.140
group:hemisphere	-0.74	2.39	39.84	-0.31	0.757
**OFA**					
(Intercept)	31.50	2.07	54.49	15.25	0.000
group (DP)	5.01	2.92	54.49	1.72	0.092
hemisphere (right)	2.14	1.64	39.97	1.30	0.201
group:hemisphere	-0.95	2.32	39.97	-0.41	0.686
**pFUS**					
(Intercept)	27.45	2.17	71.57	12.63	0.000
group (DP)	2.49	3.07	71.53	0.81	0.421
hemisphere (right)	0.54	2.49	40.03	0.22	0.828
group:hemisphere	-3.30	3.52	40.05	-0.94	0.354
**mFUS**					
(Intercept)	13.57	1.36	65.75	9.97	0.000
group (DP)	-1.15	1.92	65.83	-0.60	0.553
hemisphere (right)	-1.57	1.41	39.95	-1.12	0.271
group:hemisphere	0.45	2.00	40.10	0.23	0.822
**PPA**					
(Intercept)	28.05	1.52	60.35	18.42	0.000
group (DP)	-0.84	2.15	60.36	-0.39	0.699
hemisphere (right)	1.14	1.41	39.90	0.81	0.423
group:hemisphere	0.85	1.99	39.89	0.43	0.671

Appendix 0—table 4. Fixed effect parameter estimates for models evaluating pRF model goodness-of-fit, R^2, by hemisphere (right, left) and group (control, DP). Separate models were created for each region of interest. In each model, the intercept has been mapped to the control group in the left hemisphere.
Formula (R, lme4 package): R^2 ~ group + hemisphere + group:hemisphere + (1 | subjectID/hemisphere)
$* \ p < 0.01$; $** \ p < 0.001$
